# Wounds on the Face

**Published:** 1865-09

**Authors:** 


### Selections.

---

WOUNDS OF THL FACE.—In the *Boston Medical Journal* we find the following terse and sensible remarks by Dr. Becker:

"Wounds of the face are chiefly to be regretted on account of the deformity and disfigurement resulting therefrom. The extreme vascularity of the tissues of the face endows them with a vitality which rectifies most injuries with a rapidity truly marvellous; and from their great distensibility the surgeon is enabled to repair loss of tissue, even when this has been very extensive. The face has been wounded in almost every part and direction, and often presents a most ghastly appearance. The upper and lower jaws, respectively, have frequently been, to a greater or less extent, destroyed, and yet speedy recovery follow. At the battle of Antietam, a soldier had both eyes destroyed by one ball, which passed through the bridge of the nose, leaving a clean hole. He suffered but little pain, and made a rapid recovery.

"Hæmorrhage is undoubtedly the greatest source of danger in gunshot wounds of the face; and, from the great difficulty of commanding it, frequently places the patient in imminent danger. Those who have received a severe face wound, seldom leave the field without sustaining a considerable loss of blood; and secondary hæmorrhage is common when the bones have been fractured. The irregularity and extreme vascularity of the parts render the application of ligatures to the bleeding point difficult; and to be effectual, compresses must be applied with much nicety. In secondary hæmorrhage of the deep branches of the face, ligature of the main artery will generally be necessary.

"The branches of the facial nerve are sometimes so much injured in face wounds, either by the ball itself or by spiculæ of bone, that temporary or even permanent paralysis may ensue.

"The greatest care should always be taken to remove the secretions which result from injury of the bones of the face. For if any amount of it should be swallowed, and thus enter the stomach, much constitutional disturbance will follow, and a fever of a low typhoid and very fatal type will be iuduced.

"Fractures of the bones of the face from an exception to the general rule, of removing fragments which are nearly detached. The large supply of blood in this region frequently enables pieces of bone—whose direction is not opposed to a proper union—to resume their full connection, in a manner which would be impossible in other parts under the same relative circumstances.

"The curious manner in which balls may be concealed in the bones of the face, and be discharged of their own accord, is shown in an instance which occurred at the Alma, and is related by Macleod. 'A round ball had entered close to, but below the inner canthus of the eye, and being lost, was not further thought of. The wound healed, and the patient had almost forgotten the circumstance, when after suffering slightly from dryness in the nostril, the ball fell from his nose, to his great alarm and astonishment, several months afterwards.' This case is singular, from the absence of the fetid discharge, which usually attends such injuries of bones, with a retained ball."

